# Expression and clinical significance of CD147 in renal cell carcinoma: a meta-analysis

**DOI:** 10.18632/oncotarget.17376

**Published:** 2017-04-10

**Authors:** Hui Li, Dongwen Wu, Shupeng Shi, Yadong Xu, Ling Wei, Jing Liu, Yanting Liu

**Affiliations:** ^1^ Reproductive Department, Xiangya Hospital, Central South University, Changsha, China; ^2^ Xiangya School of Medicine, Central South University, Changsha, China

**Keywords:** renal cell carcinoma, CD147, case control study, meta-analysis

## Abstract

**Objective:**

To assess clinical significance of CD147 in renal cell carcinoma.

**Methods:**

Collect case-control studies which focus on CD147's expression in renal cell carcinoma. Trails were retrieved from CBM, CNKI, Wan-fang database, PubMed, Cochrane Library and Embase. According to the inclusion and exclusion criteria, data extraction and quality assessment were done by two researchers independently, and outcomes were pooled with Revman5.3 and STATA14.0.

**Results:**

A total of 11 studies were confirmed, among which renal cell carcinoma 887 cases, non-cancer 505cases. As for the positive rate of CD147, there are statistical differences among survival, renal cell carcinoma tissue vs. non-cancer tissues [OR= 8.19, P= 0.0002], with vs. without lymph node metastases [OR= 6.52, P= 0.001], clinical stage III~IV vs. II~I [OR= 4.07, P< 0.00001], histopathological stage III~IV vs. II [OR= 3.01, P= 0.002], histopathological stage III~IV vs. I [OR= 7.50, P< 0.00001], tumor size [OR= 5.01, P= 0.0007]. No significant difference was tested among different age, gender, histological types and Position of cancer.

**Conclusion:**

As shown in our results, CD 147 may participate the whole course of carcinogenesis of renal cell carcinoma, which might be valuable for the diagnosis, treatment and prognosis.

## INTRODUCTION

Renal cell carcinoma (RCC) is the most common malignancy of kidneys found in adults [1]. It is the seventh most common cancer in man and the tenth in women, which account for 2%~3% of all adult malignancies [2]. The incidence of RCC is increasing rapidly on average 1.1% over the last ten years [3]. In the United States, there are more than 62,000 new cases of RCC and at least 14,000 deaths expected in 2016 [2]. Tumor metastasis is the main cause of mortality and treatment failure in RCC patients [4]. Unfortunately, because of the lack of obvious symptoms during the early stage, up to 20–30% RCC patients present with metastatic disease [5]. Therefore, it is of great significance to search for sensitive and specific markers that can provide valuable information for the early diagnosis and prognosis of RCC. A number of biomarkers such as survivin, MCT1, MCT4, Cullin 1 and CD147 have been found to be involved in its development and progression according to some reports [6–8]. Among these markers, extracellular matrix metalloproteinase inducer (EMMPRIN, also known as CD147 or basigin) is highly expressed in a variety of tumors, facilitating tumor invasion and metastasis [9]. Studies show that CD147 is up-regulated in a lot of malignancies, including breast, lung, oral, esophageal, laryngeal and renal cancers [6, 10–14]. So there is great possible that CD147 can serve as a prognosis biomarker for RCC. But the expression profiles of CD147 in RCC are controversial. For example, some studies show a significant difference among the subtypes of RCC while some studies argue about this [15–18]. The same conflict is also seen in the studies of metastasis status and TNM stage [16, 18–20].

These controversies could be a result of differences in sample sizes and other factors, such as the criterial of the positive expression of CD147, and unfortunately evidence-based confirmation by large-scale clinical trials is still lacking. Therefore, we conducted this meta-analysis to quantitatively inspect the relationship between CD147 and clinicopathological features and survival of renal cancer patients.

## RESULTS

### Literature search

**Figure 1:**
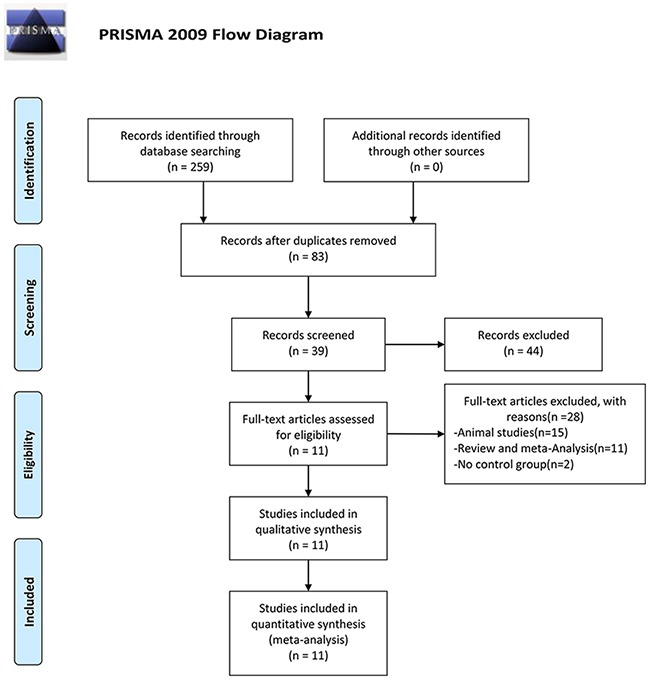
PRISMA 2009 flow diagram A total of 259 studies were identified, and 176 studies were excluded because of duplication. After reading the titles and abstracts, 44 studies were excluded. 39 possible full text studies were carefully reviewed (animal studies [n = 15]; review and meta-analysis [n = 11]; no control group [n = 2]). Finally, 11 trials were included for quantitative analysis.

A total of 259 studies were identified, and 176 studies were excluded because of duplication. After reading the titles and abstracts, 44 studies were excluded. 39 possible full text studies were carefully reviewed (animal studies [n = 15]; review and meta-analysis [n = 11]; no control group [n = 2]). Finally, 11 trials were included for quantitative analysis [15–20, 22–26] (Figure 1). Their characteristics are summarized in Table 1 and NOS score are showed in S2 Appendix.

**Table 1:** Characteristics of eligible studies

First Author	Year	Origin	Median age	Cases	Counting method	Definition of CD147 positive	Survival results	HR	follow-up time (month)	NOS score
Jin, J S[23]	2006	Taiwan	N	79	POSITIVE CELL PERCENTAGE*STAINING INTENSITY	>0% or weak intensity	OS	Univariate	70	7
Kim, Y[19]	2015	Korea	58	180	POSITIVE CELL PERCENTAGE*STAINING INTENSITY	>0% or weak intensity	OS	Univariate	173	7
Liang, Y X[22]	2009	China	63.5	53	STAINING INTENSITY	Brown	OS	Univariate	12	8
Rabien, A[16]	2013	Germany	60	394	POSITIVE CELL PERCENTAGE*STAINING INTENSITY	>0% or weak intensity	OS	Univariate	194	8
Tsai, W C[18]	2007	Taiwan	N	93	POSITIVE CELL PERCENTAGE*STAINING INTENSITY	>5% or weak intensity	OS	Univariate	70	7
Liu Z[24]	2007	China	53	80	POSITIVE CELL PERCENTAGE*STAINING INTENSITY	>0% or weak intensity	OS	Univariate	87	7
Ke M[17]	2013	China	51.2	82	STAINING INTENSITY	Brown	N	N	N	7
Wang SC[20]	2011	China	56.3	107	STAINING INTENSITY	Brown	N	N	N	8
Luo HR[15]	2016	China	56.3	85	STAINING INTENSITY	Brown	N	N	N	7
Chen YB[26]	2010	China	51.8	43	STAINING INTENSITY	Brown	N	N	N	7
Ma Y[25]	2009	China	58	50	PCR	N	N	N	N	7

positive cell percentage: 0 point for positive cell percentage≤ 25%;1 point for 26%-50%; 2 points for 51%-75%;3 points for>75%.; Staining intensity: 0 point for basically no coloration, 1 point for light yellow, 2 points for pale brown, and 3points for dark brown; N: not mention; OS: overall survival;

### CD147 expression and survival

**Figure 2:**
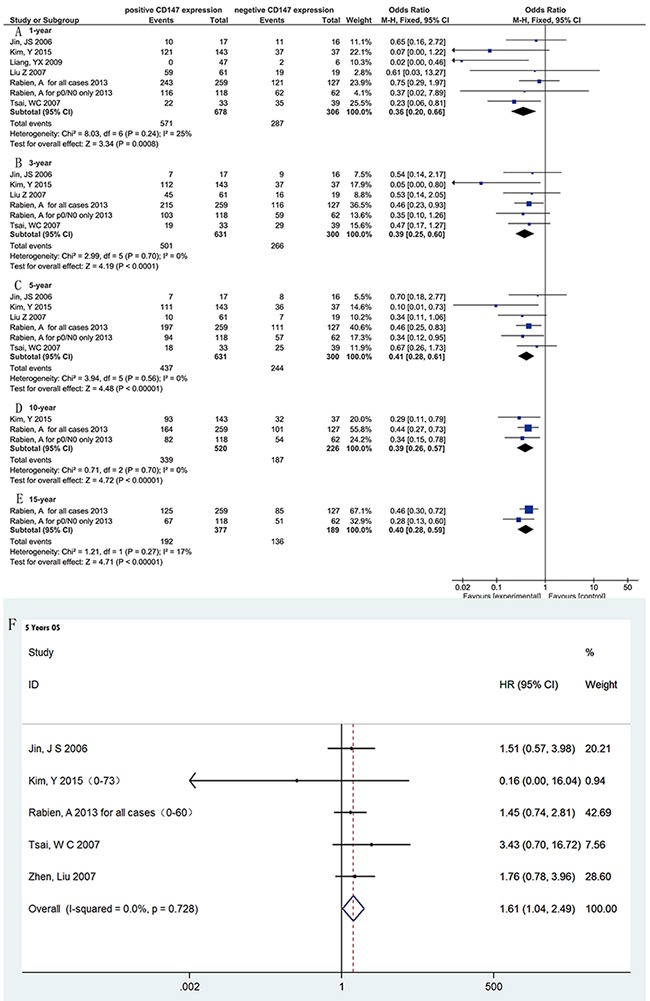
Forest Plot showing the results of meta-analyses of CD147 expression and survival **(A)** 1-year survival rate for CD 147 positive expression was worse. OR= 0.36, 95%CI= (0.20, 0.66), P= 0.0008. **(B)** 3-year survival rate for CD 147 positive expression was worse. OR= 0.39, 95%CI= (0.25, 0.60), P< 0.0001. **(C)** 5-year survival rate for CD 147 positive expression was worse. OR= 0.41, 95%CI= (0.28, 0.61), P< 0.00001. **(D)** 10-year survival rate for CD 147 positive expression was worse. OR= 0.39, 95%CI= (0.26, 0.57), P< 0.00001. **(E)** 15-year survival rate for CD 147 positive expression was worse. OR= 0.40, 95%CI= (0.28, 0.59), P< 0.00001. **(F)** 5-year HR for CD 147 positive expression was worse. HR= 1.61, 95%CI= (1.04, 2.49), P=0.015.

We investigated the relationship between CD147 expression 1-,3-,5-,10- and 15-survival. Fixed-effects model was used for all them without heterogeneity. As expected, the positive expression of CD147 indicated worse long-term survival with significant difference (Table 2, and Supplementary Figure 1). In addition, five data sets [16, 18, 19, 23, 24] with 5-year overall survival HR (hazard ratio) are avalialble, and all the studies reported the univariate result. We combined the data with fixed model effect (I2=0, P=0.728), and found that CD147 positive expression bring a worse 5-year survival comparing with negative patients (HR=1.61, 95%CI= 1.04-2.49) (Figure 2).

**Table 2:** CD147 expression and survival

Survival years	Heterogeneity		OR	95%CI	P
	P	*I^2^*			
1-year	0.24	25%	0.36	(0.20, 0.66)	< 0.0001
3-year	0.70	0%	0.39	(0.25, 0.60)	< 0.00001
5-year	0.56	0%	0.41	(0.28, 0.61)	< 0.00001
10-year	0.70	0%	0.39	(0.26, 0.57)	< 0.00001
15-year	0.27	17%	0.40	(0.28, 0.59)	< 0.00001

### CD147 in renal cancer and non-cancer tissues

**Figure 3:**
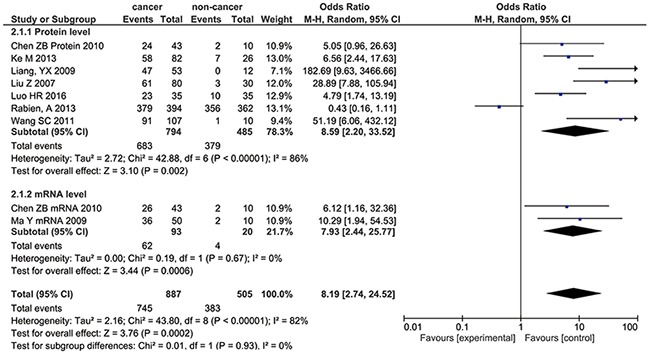
Forest Plot showing the results of meta-analyses of CD147 in renal cancer and non-cancer tissues The CD147 positive rate in renal cancer tissues was higher (OR= 8.19, 95%CI= (2.74, 24.52), P= 0.0002). Protein level (OR= 8.59, 95%CI= (2.20, 33.52), P= 0.002) and mRNA level (OR= 7.93, 95%CI= (2.44, 25.77), P= 0.0006) came to the same conclusion.

Eight studies [15–17, 20, 22, 24–26] with 1392 patients was enrolled in the positive expression of CD147 in renal cancer tissues and non-cancer tissue, which include normal tissue and para-carcinoma tissue, with 7 studies focus on protein level and 2 studies on mRNA. With significant heterogeneity (P= 0.02, I^2^= 66%), random-effects model showed the CD147 positive rate in renal cancer tissues was higher (83.99% vs. 75.84%) (OR= 8.19, 95%CI= (2.74, 24.52), P= 0.0002) (Figure 3). Subgroup analysis of different estimated level showed the same result (protein level OR= 8.59, 95%CI= (2.20, 33.52), P= 0.002; mRNA level OR= 7.93, 95%CI= (2.44, 25.77), P= 0.0006).

### CD147 expression and lymph metastasis

**Figure 4:**
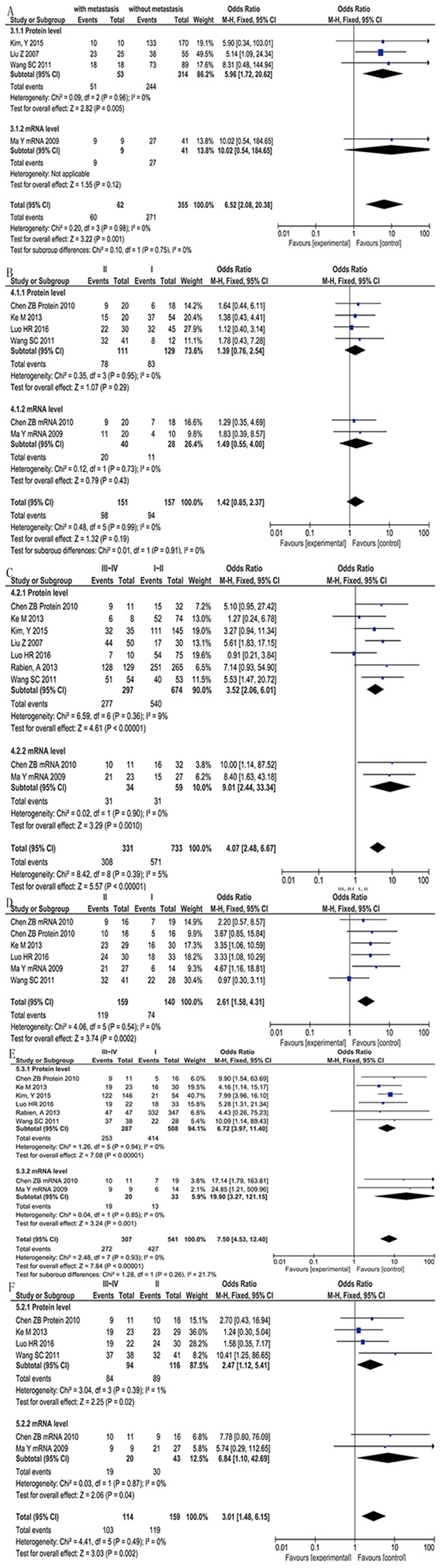
Forest Plot showing the results of meta-analyses of CD 147 Expression lymph metastasis, clinical stage and histopathological stage **(A)** CD 147 positive expression in lymph metastasis patient was more frequently than non-metastasis patient (OR= 6.52, 95%CI= (2.08, 20.38), P= 0.001). **(B)** CD 147 positive expression in TNM1 and TNM2 was no statistical difference (OR= 1.42, 95%CI= (0.85, 2.37), P=0.19). **(C)** CD 147 positive expression had a great correlation with higher TMN stage (OR= 4.07, 95%CI= (2.48, 6.67), P< 0.00001). **(D)** CD 147 positive expression in histopathological stage I vs II had significant difference (OR= 3.15, 95%CI= (1.20, 8.30), P= 0.0002). **(E)** CD 147 positive expression had a great correlation with higher histopathological stage when compared III~IV with I (OR= 7.50, 95%CI= (4.53, 12.40), P< 0.00001). **(F)** CD 147 positive expression had a great correlation with higher histopathological stage when compared III~IV with II (OR= 3.01, 95%CI= (1.48, 6.15), P= 0.002).

Four studies [19, 20, 24, 25] with 417 patients reported metastasis, including 3 studies focus on the CD147 protein level and 1 studies on mRNA. Without significant heterogeneity (P= 0.98, I^2^= 0%), fixed-effects model showed CD147 positive expression in lymph metastasis patient was more frequently than non-metastasis patient (96.77% vs. 76.34%) (OR= 6.52, 95%CI= (2.08, 20.38), P= 0.001) (Figure 4A). Protein level subgroup analysis showed the same conclusion (OR= 5.96, 95%CI= (1.72, 20.62), P= 0.005). While mRNA level subgroup showed no significant difference with only one study.

### CD147 expression and clinical stage

Totally 8 [15–17, 19, 20, 24–26] studies reported TNM Staging System (UICC,2009), some studies reported the difference of TNM1, TNM2 and TNM3-4 separately while others reported TNM1-2 and TNM3-4. No data compare the difference of TNM3 with TNM4. Therefore, firstly, we compared the difference between TNM1 and TNM2 with 6. Without heterogeneity, fixed-effect model showed there is no difference two groups (OR= 1.42, 95%CI= (0.85, 2.37), P=0.19) (Figure 4B). Then we combined TNM I and TNM II together as a group to compare with TNM III~IV with 9 date sets was available. Without heterogeneity, fixed-effects model suggested that the positive expression of CD 147 had a great correlation with higher TMN stage no matter on protein (OR= 3.52, 95%CI= (2.06, 6.01), P< 0.00001) or mRNA level (OR= 9.01, 95%CI= (2.44, 33.34), P= 0.0010) (Figure 4C).

### CD147 expression and histopathological stage

The comparision of original individual studies among different histopathological stage (The Robson Staging System of Renal Cell Carcinoma) are similar to clinical stage, so we first combined the comparing of histopathological stage I vs II, and the result had a significant difference (OR= 3.15, 95%CI= (1.20, 8.30), P= 0.0002) (Figure 4D). Therefore, we compared stage I and II with stage III~IV separately using fixed-effects model. Both of them shown that positive of CD147 were related to high histopathological stage (III~IV vs. I: OR= 7.50, 95%CI= (4.53, 12.40), P< 0.00001; III~IV vs. II: OR= 3.01, 95%CI= (1.48, 6.15), P= 0.002) (Figure 4E, Figure 4F).

### CD 147 expression with other clinicopathologic characteristics

Except for tumor size (Supplementary Figure 2A: big vs. small: OR= 5.01, 95%CI= 1.97, 12.73, P= 0.0007), there is no statistic difference between positive expression and negtive, such as age (Supplementary Figure 2B: old vs. young: OR= 1.24, 95%CI= (0.83, 1.85), P= 0.30), gender (Supplementary Figure 2C: male vs. female: OR= 0.90, 95%CI= (0.59, 1.36), P= 0.62), histological type (Supplementary Figure 2D: clear cell carcinoma vs. others: OR= 1.47, 95%CI= (0.86, 2.50), P= 0.16) and the position of tumor (Supplementary Figure 2E: higher pole vs. lower pole: OR= 1.47, 95%CI= (0.86, 2.50), P= 0.16) without heterogeneity.

### Sensitivity analysis and publication bias

We exclude study one by one to evaluate the influences of individual studies on the final effect and all the results, except 5-year survival HR, are consist with the result of including all studies (Supplementary Figure 3 sensitivity analysis), which mean our results are stable and reliable. Egger test was applied to test publication bias, publication bias did not test for almost of the result, except 4 outcomes (clinical stage II vs. I, histopathologic stage III~IV vs. II, 10-year survival and tumor size (bigger vs. small)) had a P value lower than 0.05 (Supplementary Figure 1 publication bias). By trim and fill method, both the results of fixed and random effects model are just the same with original result (S3 Appendix) (Table 3).

**Table 3:** Sensitivity Analysis and Publication bias

	OR Fluctuation	95%CI Fluctuation	Publication bias (P value)
Cancer and non-Cancer	6.41~10.88	2.16~34.04	0.142
Lympho metastasis	5.96~7.86	1.49~41.53	N
Clinical stage: III~IV vs. I~II	4.75~6.77	2.48~11.64	0.805
Histopathologic stage			
III~IV vs. I	7.12~8.31	3.55~14.43	0.689
III~IV vs. II	2.35~3.58	1.08~9.43	0.008*
Survival rate			
1-year	0.24~0.44	0.11~0.80	0,699
3-year	0.34~0.46	0.19~0.73	0.932
5-year	0.38~0.47	0.23~0.70	0.367
10-year	0.32~0.41	0.17~0.63	0.015*
15-year	0.28~0.46	0.13~0.72	N
5 year overall survival hazard ratio	1.52~1.74	0.93~3.11	0.715
Other clinicopathologic characteristics			
tumor size	3.02~5.96	1.69~18.49	0.016*
Age	1.17~1.31	0.73~2.03	0.890
Gender	0.81~1.03	0.49~1.60	0.191
Histological type	1.18~1.63	0.64~3.08	0.961
Position of cancer	0.70~0.98	0.25~3.56	N

Note: P <0.05, exist Publication Bias; N for insufficient observations.

### Bioinformatics analysis

**Figure 5:**
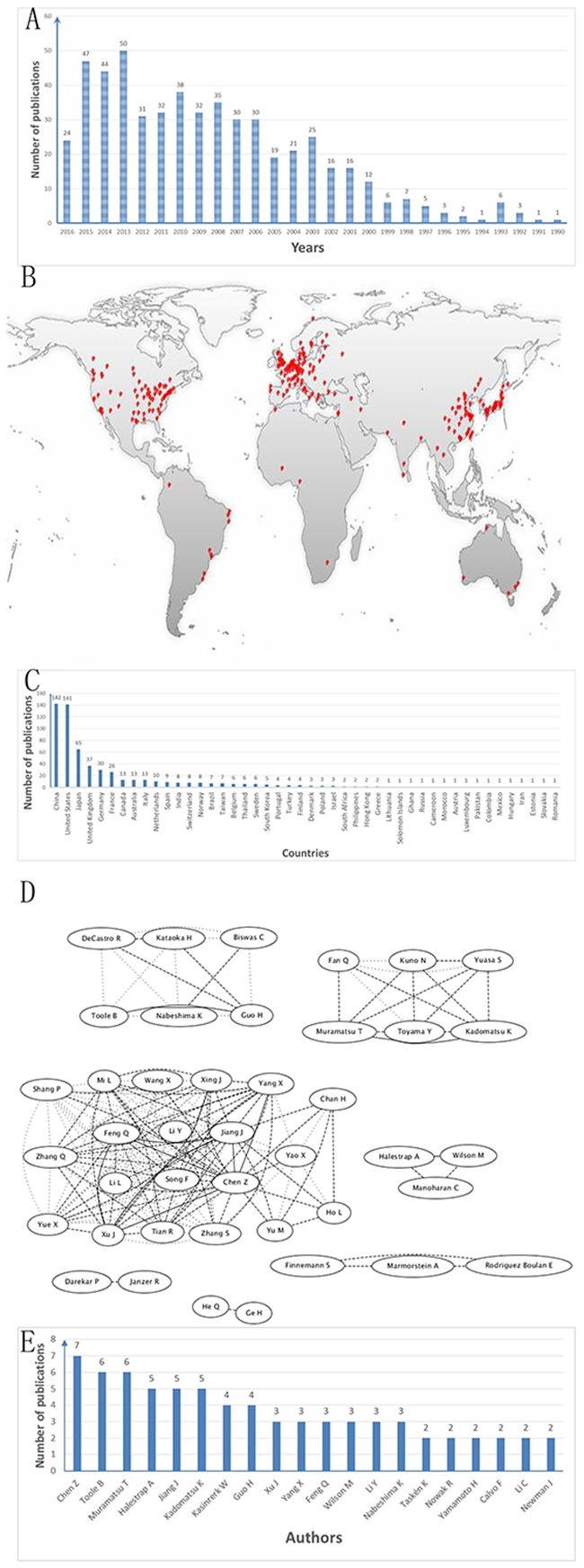
GO pubmed informatin **(A)** The discovery of CD147 was in 1990 and since then it is becoming more and more. **(B and C)** The most of research related to CD147 was investigated in China (142 publications) and the USA (141publications), followed by Japan (65 pablications). **(D)** Author networks. **(E)** The top 20 author.

**Figure 6:**
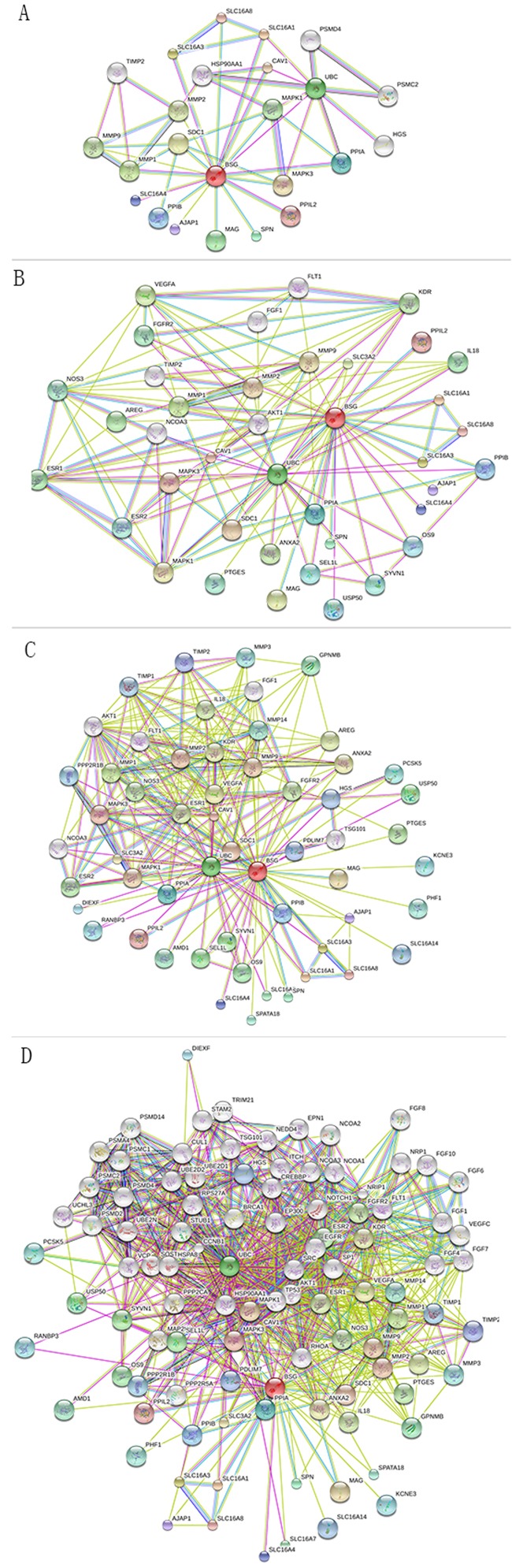
String interaction **(A)** Active interaction sources restricted only on experiment data with high confidence (0.700), max number of interactors 50 for first shell and 5 for the second shell. **(B)** High confidence (0.700) without sources restriction, max number of interactors 50 for first shell and 5 for the second shell. **(C)** Medium confidence (0.400) without sources restriction, max number of interactors 50 for first shell and 5 for the second shell. **(D)** Medium confidence (0.400) without sources restriction 50 for the first shell and the second shell

**Figure 7:**
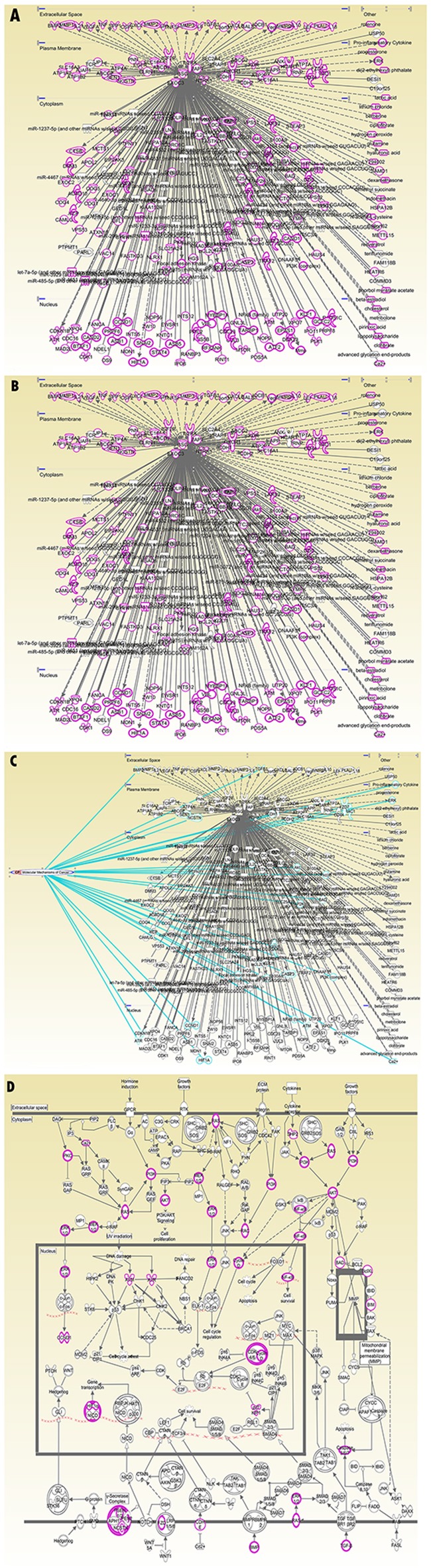
IPA interactions **(A)** Total interaction network of CD147 with other moleculars (252 moleculars) including protein and micro-RNA and their cellular location (extracellular space, plasma membrane, cytoplasma, nuleus, and other organelle) and red labeled mean the molecular is related to caner. **(B)** Red labeled in the molecular related to tumorgenesis (221/252, 87.7%). **(C)** Labeled with Blue linked line molecular is related to molecular mechanisms of cancer. **(D)** The molecular mechanism signaling pathway and the red labeled molecular is the same molecular linked with bule line in Figure 7C.

**Figure 8:**
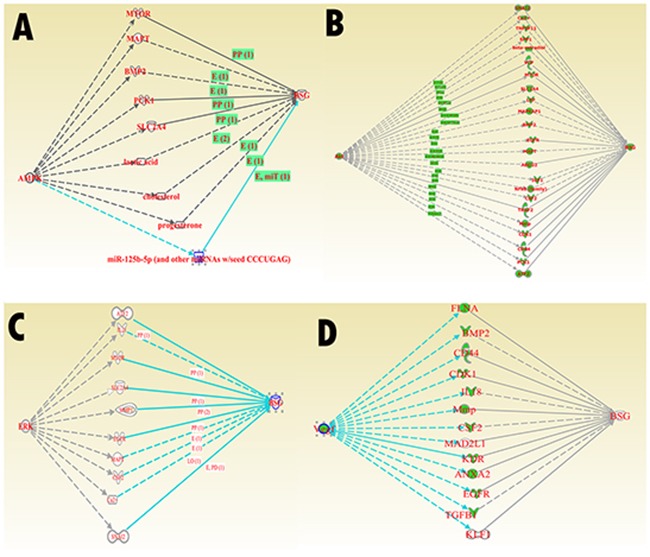
IPA core signaling molecular interaciton with CD147 **(A)** interaction between CD147 and MAPK. **(B)** interaction between CD147 and AKT. **(C)** interaction between CD147 and ERK. **(D)** interaction between CD147 and VEGF.

First, by using the GOpubmed, we explored that the discovery of CD147 was in 1990 and since then it is becoming more and more popular expecially in resecnt years (Figure 5A). In addition, most of research related to CD147 was investigated in China (142 publications) and the USA (141publications), followed by Japan (65 pablications) (Figure 5B and 5C). Besides, we can see the author networks in Figure 5D. And the top 20 author was show in Figure 5E. Second, string version10 analysis result showed the interaction between CD147 and other protiens (Figure 6A–6D). Figure 6A, active interaction sources restricted only on experiment data and B-D without sources restriction. A-B with high confidence (0.700) while C-D with medium confidence (0.400). A-C with max number of interactors 50 for first shell and 5 for the second shell, while D with 50 for the first shell and the second shell. For more details of information can be seen in supplymentary file. Third, IPA (Ingenuity PathwayAnalysis) result showed in Figure 7A–7D without species and sources restriction of the data seting. Figure 7A showed total interaction network of CD147 with other moleculars (252 moleculars) including protein and micro-RNA and their cellular location (extracellular space, plasma membrane, cytoplasma, nuleus, and other organelle) and red labeled mean the molecular is related to caner. We can know almost of all the interaction molecular is related to cancer (233/252, 92.5%). Figure 7B, red labeled in the molecular related to tumorgenesis (221/252, 87.7%). Figure 7C labeled with Blue linked line molecular is related to molecular mechanisms of cancer. And Figure 7D show the molecular mechanism signaling pathway and the red labeled molecular is the same molecular linked with bule line in Figure 7C. besides, we explored the interaction between CD147 and MAPK, AKT, ERK, VEGF which is the most core molecular in their signaling pathway (Figure 8A–8D).

.

## DISCUSSION

CD 147, also known as extracellular matrix metalloproteinase inducer (EMMPRIN), is a heavily glycosylated immunoglobulin [27]. Also there are numerous clinical study in renal carcinoma to test whether CD 147 can be a biomarker. However, the clinical relevance of CD147 remains controversial. In the current meta-analysis, we pooled the data from 13 studies together [15–20, 22–26, 35, 36], and demonstrated a remarkable association between CD147 expression of patients with renal cancer.

As shown, CD 147 is a good predictor for the survival of renal cancer patients. No matter for 1-, 3-year short term or 5-, 10-, 15-year long term survival, patients with positive expression of CD 147 did much worse than negative expression patient.

When we come to investigate the different CD 147 expression among cancer tissue and non-cancer tissue, results consist with the conclusion in other cancer [30, 32–34], which show cancer tissue is positive for CD 147 with significant statistic difference on both mRNA (OR= 7.93, 95%CI=(2.44, 25.77), P= 0.0006) and protein level (OR= 8.59, 95%CI= (2.20, 33.52), P= 0.002). So this is a good proof for CD 147 to be a potential biomarker to renal cancer.

We compared the expression of CD 147 among different lymph metastasis, clinical stage, histopathological stage, age, gender, histological type and the position of tumor to test the relationship between CD 147 and clinicopathologic characteristics. In a word, CD 147 is strongly associated with poorclinical features. Patients with lymph metastasis (OR= 6.52, P= 0.001), advanced TNM stage (OR= 4.07, P< 0.00001) and poorly differentiated histopathologic stage (III~IV vs. I: OR= 7.50, P< 0.00001; III~IV vs. II: OR= 3.01, P= 0.002) stage always have a higher expression of CD 147. However, the level of CD 147 is not bound up with age (old vs. young: P= 0.30), gender (male vs. femal P= 0.62) and position of tumor (higher pole vs. lower poleP= 0.16). Interestingly, athough renal cell carcinomas are very different among histological subtypes, we found there was no differences by comparing the CD147 positive expression between clear cell RCC and other types (P= 0.16).

CD147 has been shown to be involved in the regulation of tumor cell invasion, metastasis, angiogenesis, energy metabolism and anti-apoptosis. Firstly, by combininginteracting with integrin alpha6beta1, CD 147 take part in FAK P13K-Ca (2+) and MARK signal pathway, interstitial collagenase (MMP-1) is producted and CD147-MMP-1 complex is formed at the tumor cell surface, thus modifying the tumor cell pericellular matrix to promote invasion [37–39]. Secondly, CD147 is a receptor for platelet GPVI and mediates platelet rolling via GPVI-EMMPRIN combination, incerasing the potential metastasis [40]. Thirdly, it can influence the VEGF/VEGF receptor system of endothelial cells and increase the blood supply for tumor [41]. For glycolytic energy metabolism, CD147 promote the lactate transportion to feed the tumor with the help of AMPK and MCT1/2 [42, 43]. Last but not least, CD147 can down-regulate Beclin 1 and inhibit starvation-induced autophagy through the PI3K/Akt/mTOR pathway, modulating the apoptosis of tumor [44, 45]. And our results demonstrated that poor clinical performance for renal cancer patients, such as lymph metastasis, higher TMN and histopathologic stage, bigger tumor size, is usually accompanied by positive CD 147. So the survival time for them would be much shorter than CD 147 negetive patients.

Efforts were made to conduct a comprehensive analysis, but some limitations need to be acknowledged. First, although we have tried but no unpublished data was found, so all included studies were published data. Publication bias existed in 10-year survival and the tumor size. But trim and fill analysis (S3 Appendix) showed the same results which means the result is reliable. Second, survival analysis was not performed by multivariate analyses in most reported studies, this could bring some bias. Third, external virtuality was limited as most included patients were Chinese. Last, the definition of CD147 positive expressin criteria in all studies isn't unified, which could bring some potential bias, fortunately, the sensitivity analyses showed a stable result.

To our knowledge, this meta-analysis is the first study which systematically estimates the association between CD147 expression and the survival analysis and its clinicopathological parameters. Nowadays, early diagnosis and earlytreatment are the fundamental approaches to improve prognosis [46]. Our results found that CD147 positive expression was significant associated with renal caner tissues, which supported that CD147 could be applied as a potential clinical marker for the early diagnosis of renal cancer. In our study, we demonstrated that CD147 positive expression strongly predicted poorer TNM stage, histopathologic stage, lymph node metastasis and worse survival in the patients with renal cancer. Further studies using additional putative renal cancer surface markers in combination with CD147 are required to evaluate their potential use in predicting patients’ outcome.

## MATERIALS AND METHODS

### Search strategy

We searched PubMed (1966-2016), EMBASE (1980-2016), the Cochrane Library (1996-2016), Web of Science (1945-2016), China National Knowledge Infrastructure (1982-2016), and the WanFang databases (1988-2016). The studies were restricted to humans, but not restricted by date, language, or publication status. The following combined search term was used: (kidney neoplasms, renal neoplasms, kidney tumor, renal tumor, kidney cancer, renal cancer, kidney carcinoma, renal carcinoma, clear cell carcinoma) AND (CD147, extracellular matrix metalloproteinase inducer, EMMPRIN, BSG). We combined the term appropriately with MeSH Terms and used an appropriate adjustment for different databases. Details of the search strategies can be found in S1 Appendix.

### Criteria for including studies

1. Published or unpublished case control study or cohort study in English or Chinese with the full text available;
2. Cases have survival data or clinical pathological characteristic data, without radiotherapy or chemotherapy or biological therapy before sampling;
3. Pathological methods for confirming renal cancer.
4. CD147 expression based on primary renal cancer tissue, rather than serum or any other kinds of indirect specimen were included.
5. Choosing best quality study for duplication.

### Criteria for excluding studies

1. Cell or animal studies, case reports, letters, reviews.
2. The standard of pathological diagnosis was not clear;

### Statistical analysis

Bibliographies were scanned by two authors independently to exclude unrelated studies. Then, full text were reviewed, data were extracted independently and controversy were solved by disscussion. The software Revman 5.3 and Stata 14.0 were applied to analyze the data. Results were showed with odds ratios (OR) or standard mean difference (SMD) and 95% confidence intervals (95% CI). Fixed-effects model was adopted for non-heterogeneity data (P > 0.1 and I^2^ < 50%); otherwise, random-effects model. If possible, heterogeneity subgroup analyses were performed. All P values were 2-sided, and P < 0.05 was considered significant. Sensitive analysis was also performed to evaluate the influences of individual studies on the final effect. Egger's test was used to assess publication bias (P < 0.05 was considered statistically significant). If publication bias was confirmed, a trim-and-fill method developed by Duval and Tweedie was implemented to adjust for this bias [21]. Then, we replicated the funnel plot with their ‘‘missing’’ counterparts around the adjusted summary estimate.

### Bioinformatics analysis

Firstly, we used GOpubmed (http://www.gopubmed.com/web/gopubmed/) with the Search term““Antigens, CD147“[mesh]”to get information about the current related published studies with a global perspective. Secondly, we applied String vesrion10 (http://string-db.org/) to explore the interaction between CD147 and other moleculars. We choosed the organism with Homo sapiens, and mathed the information with different minimum required interaction score, active interaction sources and max number of interactors. Thirdly, IPA (Ingenuity PathwayAnalysis) (http://www.ingenuity.com/) was employed to uncover the interaction network between CD147 and other moleculars with different threshold without species restriction. And IPA pathyway explorer was used to explore some important signaling pathways which CD147 has take parted in.

## SUPPLEMENTARY MATERIALS FIGURES AND TABLES







## Abbreviations

CNKI: Chinese periodical full text database
CBM: China Biology Medicine disc
RCC: Renal cell carcinoma
EMMPRIN: extracellular matrix metalloproteinase inducer
OR: odds ratios
MCT: monocarboxylate transporter
HR: hazard ratio
CI: confidence intervals
OS: overall survival
IPA: Ingenuity PathwayAnalysis
GPVI: Glycoprotein VI
VEGF: vascular endothelial growth factor
BSG: Basigin
SMD: standard mean difference
