# Supplementary material for: Expression and clinical significance of CD147 in renal cell carcinoma: a meta-analysis
Source: Oncotarget. 2017 Apr 10;8(31):51331–44. doi: 10.18632/oncotarget.17376 (PMC5584252; doi:10.18632/oncotarget.17376)
Supplement: Supplementary file 1 [file oncotarget-08-51331-s001.pdf]

Expression and clinical significance of CD147 in renal cell carcinoma: a meta-analysis

SUPPLEMENTARY FIGURES

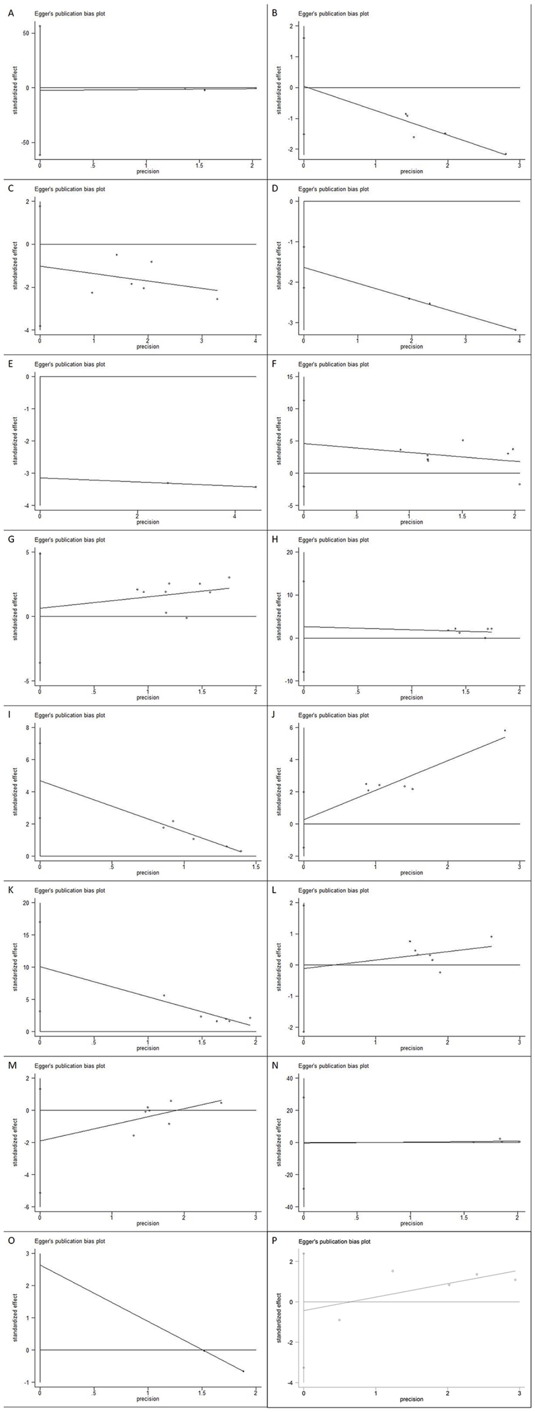

Supplementary Figure 1: Publication bias. Except clinical stage II vs. I, histopathologic stage III~IV vs. II, 10-year survival and tumor size (bigger vs. small), ohters all had a P value higher than 0.05.

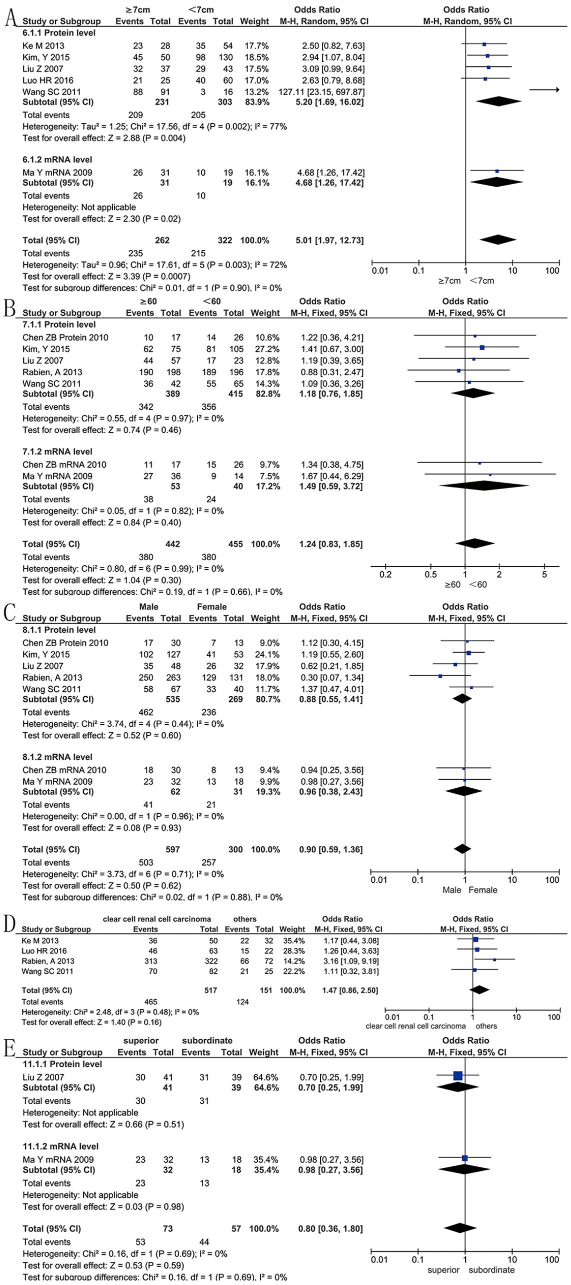

Supplementary Figure 2: Forest Plot showing the results of meta-analyses of CD147 expression with other clinicpathological charicteristicsothers. (A) CD 147 positive expression in different tumor size had statisrical difference (big vs. small: OR= 5.01, 95%CI= 1.97, 12.73, P= 0.0007). **(B)** CD 147 positive expression in different age group had no statisrical difference (old vs. young: OR= 1.24, 95%CI= (0.83, 1.85), P= 0.30). **(C)** CD 147 positive expression in different gender had no statisrical difference (male vs. female: OR= 0.90, 95%CI= (0.59, 1.36), P= 0.62). **(D)** CD 147 positive expression in different histological type had no statisrical difference (clear cell carcinoma vs. others: OR= 1.47, 95%CI= (0.86, 2.50), P= 0.16). **(E)** CD 147 positive expression in different position of tumor had no statisrical difference (higher pole vs. lower pole: OR= 1.47, 95%CI= (0.86, 2.50), P= 0.16).

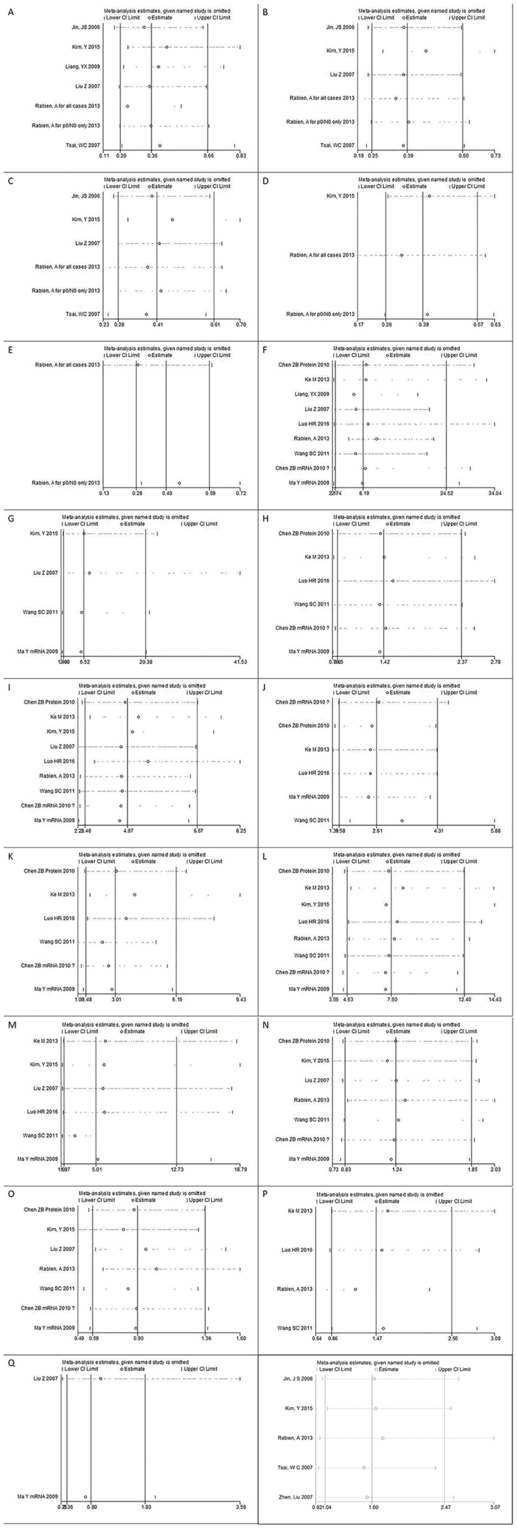

Supplementary Figure 3: Sensitivity analysis. Except 5-year survival HR, others are consist with the result of including all studies.

Appendix 1: Retrieval strategy.

See Supplementary File 1

Appendix 2: NOS score

**Column**	**Entries**	**First author**										
		1	2	3	4	5	6	7	8	9	10	11
Section	Is the definition adequate	☆	☆	☆	☆	☆	☆	☆	☆	☆	☆	☆
	Representativeness of the cases	☆	☆	☆	☆	☆	☆	☆	☆	☆	☆	☆
	Selection of controls	☆	☆	☆	☆	☆	☆	☆	☆	☆	☆	☆
	Definition of controls	☆	☆	☆	☆	☆	☆	☆	☆	☆	☆	☆
Comparability	Comparability of cases and controls on the basis of the design and analysis	☆	☆	☆	☆	☆	☆	☆	☆	☆	☆	☆
	Ascertainment of exposure	☆	☆	☆	☆	☆	☆	☆	☆	☆	☆	☆
Exposure	Same method of ascertainment for cases and controls	☆	☆	☆	☆	☆	☆	☆	☆	☆	☆	☆
	Non-Response rate	☆	☆	☆	☆	☆	☆	☆	☆	☆	☆	☆
Total scores	2016	7	7	8	8	7	7	7	8	7	7	7

Notes: 1. Jin, J S; 2. Kim, Y; 3. Liang, Y X; 4. Rabien, A; 5. Tsai, W C; 6. Liu Z; 7. Ke M; 8. Wang SC; 9. Luo HR; 10. Chen YB; 11. Ma Y.

Appendix 3: Tirm and fill analysis for bias data.

See Supplementary File 2
