# Supplementary material for: Expression and clinical significance of CD147 in renal cell carcinoma: a meta-analysis
Source: Oncotarget. 2017 Apr 10;8(31):51331–44. doi: 10.18632/oncotarget.17376 (PMC5584252; doi:10.18632/oncotarget.17376)
Supplement: Supplementary file 2 [file oncotarget-08-51331-s002.docx]

Retrieval result

Pubmed: 53

Embase: 68

Cochrane Library: 0

WOS: 56

CNKI: 36

WANFANG: 46

Pubmed

antigens, cd147[MeSH Terms] OR cd147[Title/Abstract] OR (extracellular[Title/Abstract] AND matrix[Title/Abstract] AND metalloproteinase[Title/Abstract] AND inducer[Title/Abstract]) OR "extracellular matrix metalloproteinase inducer"[Title/Abstract] OR EMMPRIN[Title/Abstract]

Kidney Neoplasms [MeSH Terms] OR (Kidney [Title/Abstract] AND Neoplasms[Title/Abstract]) OR (Kidney[Title/Abstract] AND tumor[Title/Abstract]) OR Kidney[Title/Abstract] OR renal [Title/Abstract]

Embase

(('CD147 antigen'/exp OR cd147:ti,ab OR (extracellular:ti,ab AND matrix:ti,ab AND metalloproteinase:ti,ab AND inducer:ti,ab) OR "extracellular matrix metalloproteinase inducer":ti,ab OR EMMPRIN:ti,ab) AND (' Kidney Neoplasms'/exp OR Kidney tumor:ti,ab OR Kidney Neoplasms:ti,ab OR Kidney:ti,ab OR renal:ti,ab)) AND [embase]/lim

Cochrane Library

(Kidney Neoplasms:ti,ab or Kidney tumor:ti,ab or Kidney:ti,ab or renal:ti,ab) and ([mh "Antigens, CD147"] or CD147:ti,ab or (extracellular:ti,ab and matrix:ti,ab and metalloproteinase:ti,ab and inducer:ti,ab) or "extracellular matrix metalloproteinase inducer":ti,ab or EMMPRIN:ti,ab)

WOS

(TS=Antigens, CD147 OR TS=cd147 OR (TS=extracellular AND TS=matrix AND TS=metalloproteinase AND TS=inducer) OR TS="extracellular matrix metalloproteinase inducer" OR TS=EMMPRIN) AND (TS= Kidney Neoplasms OR TS= Kidney tumor OR TS= Kidney OR TS= renal)

CNKI

SU='CD147' OR SU='抗原, CD147' OR SU='细胞外基质金属蛋白酶诱导因子'

SU='肾癌' OR SU='肾'

WANFANG

(主题:(CD147) + 主题:(抗原, CD147) + 主题:(细胞外基质金属蛋白酶诱导因子)) * （主题:(肾癌) + 主题:(肾)）
