# Supplementary material for: Expression and clinical significance of CD147 in renal cell carcinoma: a meta-analysis
Source: Oncotarget. 2017 Apr 10;8(31):51331–44. doi: 10.18632/oncotarget.17376 (PMC5584252; doi:10.18632/oncotarget.17376)
Supplement: Supplementary file 3 [file oncotarget-08-51331-s003.docx]

Clinical stage III~IV vs. I~II

| Pooled 95% CI Asymptotic No. of

Method | Est Lower Upper z_value p_value studies

-------+----------------------------------------------------

Fixed | 0.285 -0.224 0.794 1.097 0.273 6

Random | 0.285 -0.224 0.794 1.097 0.273

| Pooled 95% CI Asymptotic No. of

Method | Est Lower Upper z_value p_value studies

-------+----------------------------------------------------

Fixed | 0.115 -0.317 0.547 0.522 0.602 9

Random | 0.115 -0.317 0.547 0.522 0.602

Histopathologic stage III~IV vs. II

| Pooled 95% CI Asymptotic No. of

Method | Est Lower Upper z_value p_value studies

-------+----------------------------------------------------

Fixed | 0.918 0.141 1.694 2.315 0.021 5

Random | 0.918 0.140 1.696 2.314 0.021

| Pooled 95% CI Asymptotic No. of

Method | Est Lower Upper z_value p_value studies

-------+----------------------------------------------------

Fixed | 0.487 -0.208 1.181 1.373 0.170 7

Random | 0.503 -0.412 1.418 1.078 0.281

Survival 10-year

| Pooled 95% CI Asymptotic No. of

Method | Est Lower Upper z_value p_value studies

-------+----------------------------------------------------

Fixed | -0.938 -1.332 -0.543 -4.656 0.000 3

Random | -0.938 -1.332 -0.543 -4.656 0.000

| Pooled 95% CI Asymptotic No. of

Method | Est Lower Upper z_value p_value studies

-------+----------------------------------------------------

Fixed | -0.938 -1.332 -0.543 -4.656 0.000 3

Random | -0.938 -1.332 -0.543 -4.656 0.000

tumor size

| Pooled 95% CI Asymptotic No. of

Method | Est Lower Upper z_value p_value studies

-------+----------------------------------------------------

Fixed | 1.412 0.923 1.901 5.661 0.000 6

Random | 1.612 0.679 2.544 3.388 0.001

| Pooled 95% CI Asymptotic No. of

Method | Est Lower Upper z_value p_value studies

-------+----------------------------------------------------

Fixed | 1.412 0.923 1.901 5.661 0.000 6

Random | 1.612 0.679 2.544 3.388 0.001
